# Sarcopenic obesity is significantly associated with poorer overall survival after liver transplantation: a systematic review and meta-analysis

**DOI:** 10.3389/fnut.2024.1387602

**Published:** 2024-12-16

**Authors:** Hui-Bin Huang, Yi-Bing Zhu, Da-Xing Yu

**Affiliations:** Department of Critical Care Medicine, Guang'anmen Hospital, China Academy of Chinese Medical Sciences, Beijing, China

**Keywords:** sarcopenic obesity, liver transplantation, meta-analysis, survival, obesity

## Abstract

**Background:**

Sarcopenia has been shown to worsen survival after liver transplantation. However, it remains unclear whether coexisting sarcopenia and obesity, so-called sarcopenic obesity (SO), may also synergistically increase their adverse effects. This meta-analysis aimed to evaluate whether pre-transplant SO independently predicts survival in this population.

**Methods:**

We conducted this study according to the Preferred Reporting Items for Systematic Review and Meta-Analyses guidelines. The PubMed, Embase, Web of Science, Wanfang, CNKI, and Cochrane databases were searched up to 15 October 2023, for studies with any study design evaluating the relationship between SO and post-transplant survival in patients undergoing liver transplantation. We used ROBINS-E to assess the study quality. The primary outcome was all-cause mortality at any length of follow-up. We calculated pooled odds risks (ORs) or hazard risks (HRs) with 95% confidence intervals (CIs). Heterogeneity was quantified with *I*^2^ statistics. Subgroup analyses and publication bias evaluations were also conducted.

**Results:**

We included nine cohort studies with 2,416 patients. These studies were moderate to high quality. Pre-liver transplant patients commonly experience SO, with a mean prevalence as high as 34%. Overall, patients with SO exhibited a significantly higher overall mortality than patients without SO, as demonstrated by pooled studies using both univariate analysis [HR = 1.76, 95%C 1.33–2.33, *p* < 0.0001] and multivariate analysis (HR = 2.33, 95%CI 1.34–4.04, *p* = 0.003). Similar results were also found when comparing patients with or without SO at 1, 3, and 5 years of follow-up (OR = 1.86, 95%CI 1.22–2.83; OR = 1.83, 95%CI: 1.27–2.64; and OR = 1.54, 95% CI 1.02–2.34, respectively). In addition, subgroup analysis based on studies that reported HRs of both sarcopenia and SO indicated both had independent negative effects on post-transplant survival.

**Conclusion:**

Our meta-analysis showed that SO occurs frequently in liver transplant patients. SO is associated with an increased risk of mortality in such patient populations.

**Systematic review registration:**

https://doi.org/10.37766/inplasy2024.2.0069 [inplasy2024.2.0069].

## 1 Introduction

Sarcopenia was first defined in 1989 as the loss of skeletal muscle mass and strength (1). Initially recognized as an age-related skeletal muscle atrophy, it has since been observed in various clinical diseases (2–4). Among these, pre-liver transplantation (LT) patients often suffer from sarcopenia due to various diseases (eating disorders, chronic disease wasting, and anticancer treatments) and demographic conditions (advanced age, obesity, and activity limitations) (5). Numerous studies have shown that pre-LT sarcopenia is strongly associated with poor survival, longer hospital stays, more postoperative complications, and increased healthcare costs (6, 7).

Obesity, as an underlying condition that can lead to many diseases and pathologies, is frequently associated with diabetes mellitus, abnormal lipid metabolism, hypertension, and non-alcoholic fatty liver disease (8). It has been shown to increase the risk of anesthesia and surgical complications and is considered a predictor of perioperative outcomes (9–11).

Sarcopenic obesity (SO), which combines sarcopenia and obesity and may lead to a higher risk of adverse outcomes than either muscle loss or obesity alone (12), is receiving increasing attention in the field of LT. The North American Liver Transplantation Sarcopenia Working Group guidelines on sarcopenia in LT, developed in 2019, stated that patients with sarcopenia should be the focus of research in LT (7). However, few studies have addressed the prognostic value of SO in this patient population. A previously published meta-analysis suggested that SO might worsen survival after LT (13). However, the inclusion of data from only three studies hinders the full interpretation of their results. On the other hand, the obesity paradox, the U-shaped relationship between mortality and obesity as defined by body mass index, may influence the performance characteristics of SO (14). Among elderly patients with SO, obesity is protective against functional status impairment (14). In addition, variations in the definitions and cutoffs for SO, as well as the use of different imaging techniques (such as computed tomography and magnetic resonance imaging), can cause widely varying prevalence rates of sarcopenia and affect the reliability of prognostic assessments (15). To reconcile these findings and strengthen the role of SO as a potential prognostic factor in LT patients, an updated systematic review and meta-analysis of the existing evidence is both timely and necessary.

Recently, several relevant studies on this topic have emerged (16–19). To obtain a more reliable evaluation, we aimed to investigate the impact of SO on survival in patients with LT.

## 2 Methods

### 2.1 Protocol and registration

We followed the Preferred Reporting Items of Systematic Reviews and Meta-Analyses (PRISMA) checklist to prepare this review (20) (Supplementary File 1). The review protocol for this study was prospectively registered and published via the International Platform of Registered Systematic Review and Meta-analysis Protocols database.^1^

### 2.2 Search strategy and selection criteria

Two authors (H-BH and Y-BZ) independently searched the PubMed, EMBASE, Web of Science, Wanfang, and the China National Knowledge Infrastructure (CNKI) databases, and the Cochrane Library for eligible studies up to 16 October 2023. The search terms used were “sarcopenia AND obesity AND liver transplant” with no language restriction. In addition, the gray literature^2^ was also searched. The details of the search strategy are summarized in Supplementary File 2. After importing the studies into Endnote to exclude duplicates, we searched the literature for relevant articles by screening titles, abstracts, and full texts. We also screened the reference lists of the included articles and previous reviews to identify other potentially eligible studies. Discrepancies were resolved by discussion between the two authors or by a third author (D-XY).

Study inclusion criteria were as follows: (1) study design: any cohort study; (2) study population: adult (>18 years) patients receiving LT; (3) intervention: patients with SO (defined by the authors) compared to those without SO (NSO) or non-sarcopenic non-obese (NN) patients; and (4) predefined outcomes: the main outcome was all-cause mortality at any length of follow-up, regardless of reporting type. When the same cohort was reported in multiple publications, we retained only the most informative article or completed study to avoid duplication of information.

We excluded the studies that focused on children or pregnant women, or that lacked clear survival information. Studies available only as comments, abstracts, reviews, meta-analyses, or meeting reports were also excluded.

### 2.3 Data extraction and quality assessment

Two authors (H-BH and Y-BZ) independently extracted data from included studies as follows: first author, year of publication, sample size, study design, country, follow-up, basic patient demographics (age, gender, and body mass index [BMI]), definitions and incidences (sarcopenia, obesity, and SO), CT methods (location of scan and time point of exam), and outcome data. Disagreements were resolved by discussion and consensus.

We evaluated the study quality using the Risk Of Bias In Non-randomized Studies - of Exposure (ROBINS-E) (21). The ROBINS-E is made up of seven domains, and the assessment of the risk of bias for each domain is categorized as low, some concerns, or high. The overall risk of bias assessment of CTs was determined by the category of highest risk of bias among seven domains. For concerns not applicable to the studies, we assumed that there were no domain issues. Discrepancies were identified and resolved through discussion.

### 2.4 Statistical analysis

We combined the results of all relevant studies to estimate pooled odds ratios (ORs) and associated 95% confidence intervals (CIs) for dichotomous outcomes and mean differences (MDs) and 95% CIs for continuous outcomes. We calculated ORs and 95% CIs for studies that reported mortality rates between patients with and without myosteatosis. For studies that used regression analysis to investigate the relationship between myosteatosis and mortality, we used the inverse variance method to combine mortality estimates with corresponding standard errors. Thus, ORs or hazard ratios (HRs) reported in these studies required natural logarithmic transformation before merging. Unless otherwise noted, we preferred to use adjusted analysis results.

We examined the heterogeneity among these studies using the *I*^2^ statistic, with low, moderate, high, and substantial heterogeneity defined as *I*^2^ < 25%, *I*^2^ = 25–50%, *I*^2^ = 50–77%, and *I*^2^ > 75%, respectively. We chose fixed-effect models for *I*^2^ < 25% and random-effect models for I2 ≥ 25% (22). To explore the potential confounding factors, we conducted sensitivity analyses by excluding one study at a time to determine whether any individual study influenced the overall results. We also conducted subgroup analyses of the primary outcome by pooling studies reporting different obesity definitions [i.e., BMI vs. visceral fat area (VFA)]. Before data analysis, we estimated the mean from the median and the standard deviations (SDs) from the IQR using the methods of the previous study (23). We assessed publication bias by visually exploring funnel plots for asymmetry. We used Review Manager version 5.4 for all analyses.

## 3 Results

### 3.1 Study selection

**Figure 1:**
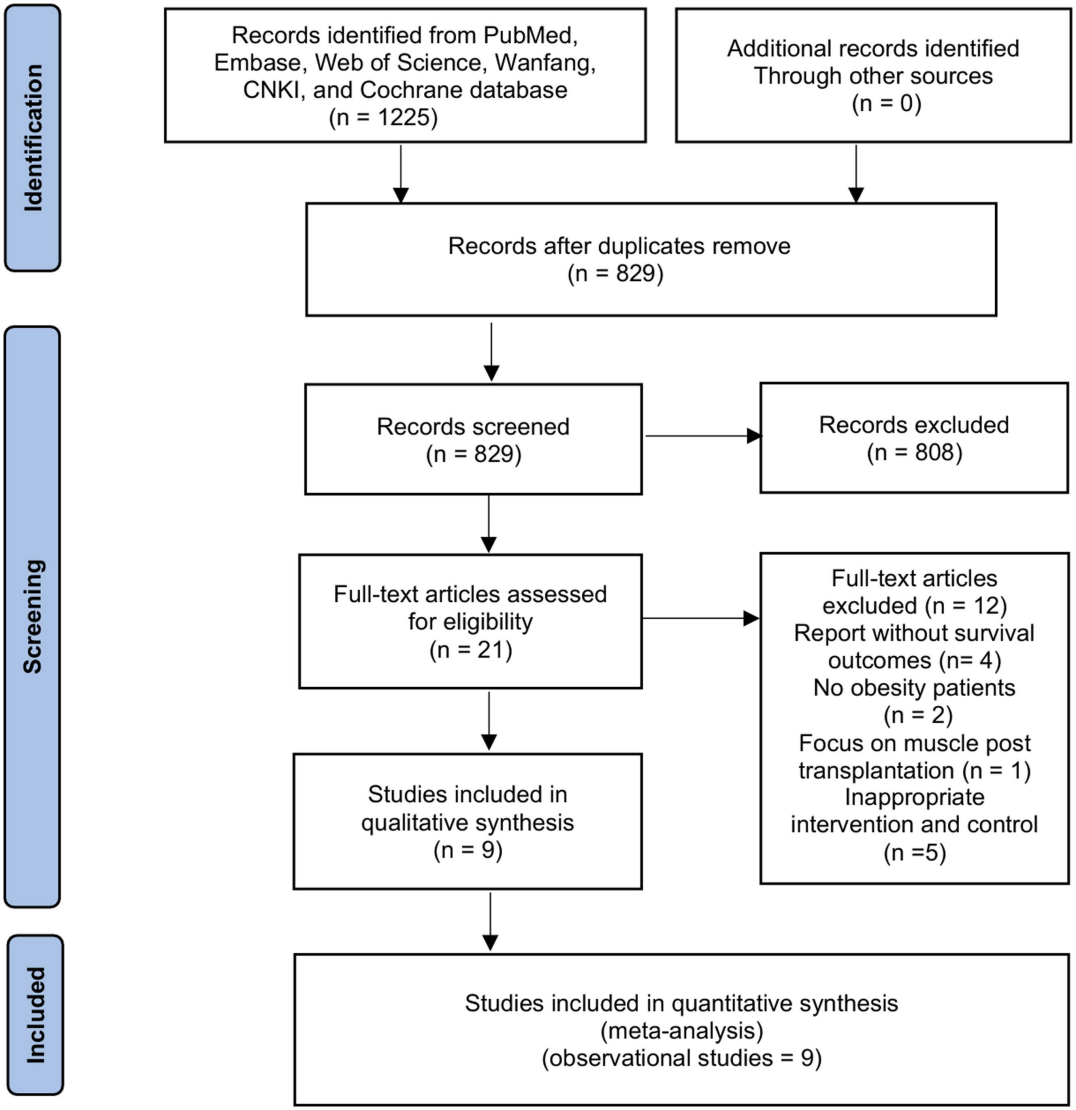
Selection process for the studies included in the meta-analysis.

The initial search identified 1,225 papers (Figure 1). The review of titles and abstracts excluded 1,204 papers, leaving 21 studies for full-text review. Finally, we included nine retrospective cohort studies with 2,416 recipients in our systemic review and meta-analysis (16–19, 24–28).

### 3.2 Characteristics and methodological quality

Table 1 summarizes the main characteristics of the included studies, the methods used for muscle assessment methods (i.e., measured tools and evaluated location), and the definition and prevalence of sarcopenia, obesity, and SO. Of the included studies, five defined SO as the coexistence of sarcopenia and a BMI ≥25 kg/m^2^ (16, 17, 24, 27, 28), three defined SO as reduced muscle mass and increased visceral fat area on CT (18, 19, 26), and one study used both definitions (25).

**Table 1:** Study characteristics of the included studies.

Author	Country	Design	Sample, N/SO	Age, mean year, N/SO	Male, %N/SO,	BMI, kg/m^2^	MELD	Study period	Longest follow-up	ROBINS -E	Measured location	Definition of sarcopenia	Sarcopenia, %	Obesity definition	Obesity, %	Definition of sarcopenic obesity	SO, %
Carias et al. (28)	USA	R, SC	207/38	54/	68.6/	30.1	21	2008–2013	5 years	Low	CT: L3	SMI ≤52.4 cm^2^/m^2^ in males and ≤ 38.5 cm^2^ /m^2^ in females	108 (52.1)	BMI >30 kg/m^2^	101 (48.8)	Defined as obesity (BMI >30) in the setting of sarcopenia	38 (18.4)
Czigany et al. (19)	Germany	R, SC	225/34	55/	52/	30	20	2010–2017	90 days	Moderate	CT: L3	SMI < 50 cm^2^/m^2^ for men and < 39 cm^2^/m^2^ for women	84 (37.3)	VFA	137 (60.9)	The combination of reduced SMI and high VFA ≥ 100 cm^2^	34 (15.1)
Ha 2022 (27)	USA	R, MC	116/23	53/54	76/78	27.5	30	2005–2017	3 years	Low	CT: L3	SMI < 50 cm^2^/m^2^ for men and < 39 cm^2^/m^2^ for women	52 (44.8)	BMI ≥25 kg/m^2^, VAT	49 (24.5)	Using CT-based SMI and visceral-to-subcutaneous adipose tissue ratio ≥ 1.54 in men and ≥ 1.37 in women	23 (19.8)
Ham et al. (18)	Japan	R, SC	200/10	42/42.6	56/70	23.3	21.5	2008–2013	5 years	Moderate	CT: umbilical level;	PMI of <6.36 cm^2^/m^2^ for men or < 3.92 cm^2^/m^2^ for female	71 (35.5)	BMI ≥25 kg/m^2^	59 (29.5)	The coexistence of sarcopenia and BMI ≥25 kg/m^2^	10 (5)
Irwin et al. (17)	South Africa	R, SC	106/36	50/52	60.4/77.8	NA	NA	2011–2019	1 year	Moderate	CT: L3	SMI of <39 cm^2^/m^2^ for women and < 50 cm^2^/m^2^ for men	69 (65)	BMI ≥25 kg/m^2^	65 (61.3)	The coexistence of sarcopenia and BMI ≥25 kg/m^2^	36 (34)
Itoh et al. (26)	Japan	R, SC	153/38	58/57	56.2/78.9	23.9	NA	2001–2012	10 years	Moderate	CT: umbilical level; L3	According to SMI	NA	VFA	NA	Simultaneous severe obesity and low SMI.	38 (24.8)
Kamo et al. (25)	Japan	R, SC	277/6	54/57	48.4/70	20.9	17	2008–2016	10 years	Low	CT: L3	SMI of <40.3 cm^2^/m^2^ for women and < 30.88 cm^2^/m^2^ for men	54 (19.5)	BMI ≥25 kg/m^2^, VFA	49 (17.7)	Combination of low SMI and either VFA >100 cm^2^ or BMI ≥25 kg/m^2^	9 (3)
Montano-loza et al. (24)	Canada	R, SC	678/135	57/58	67/83.7	27.4	15	2000–2013	2 years	High	CT: L3	SMI: ≤41 cm^2^/m^2^ for women and ≤ 53 cm^2^/m^2^ for men with BMI ≥25 and ≤ 43 cm^2^/m^2^ with BMI <25	292 (38.6)	BMI ≥25 kg/m^2^	419 (61.8)	Defined as concurrent sarcopenia and overweight or obesity	135 (19.9)
Shafaat et al. (16)	USA	R, SC	454/29	57/	64.8/	29	21.4	2009–2018	10 years	Moderate	CT: L3	SMI ≤ 50 cm^2^/m^2^ for men and ≤ 39 cm^2^/m^2^ for women	136 (30.0)	BMI ≥30 kg/m^2^	NA	Defined as concurrent obesity (BMI > 30) and sarcopenia	29 (6.4)

BMI, body mass index; MELD, model for end-stage liver disease; MRI, magnetic resonance imaging; N, number of total studies; NA, not available; R, retrospective; ROBINS-E, Risk of bias in nonrandomized studies of exposures; SC, single-center; SMI, skeletal muscle index; SO, sarcopenic obesity.

The risk of assessment is presented in Supplementary File 3 and shows that various quality assessments ranged from low to high. Three studies had a low risk of bias across all the domains, and the remaining studies were judged poorly due to confounding and measurement of outcomes.

### 3.3 Sarcopenic obesity and mortality

**Figure 2:**
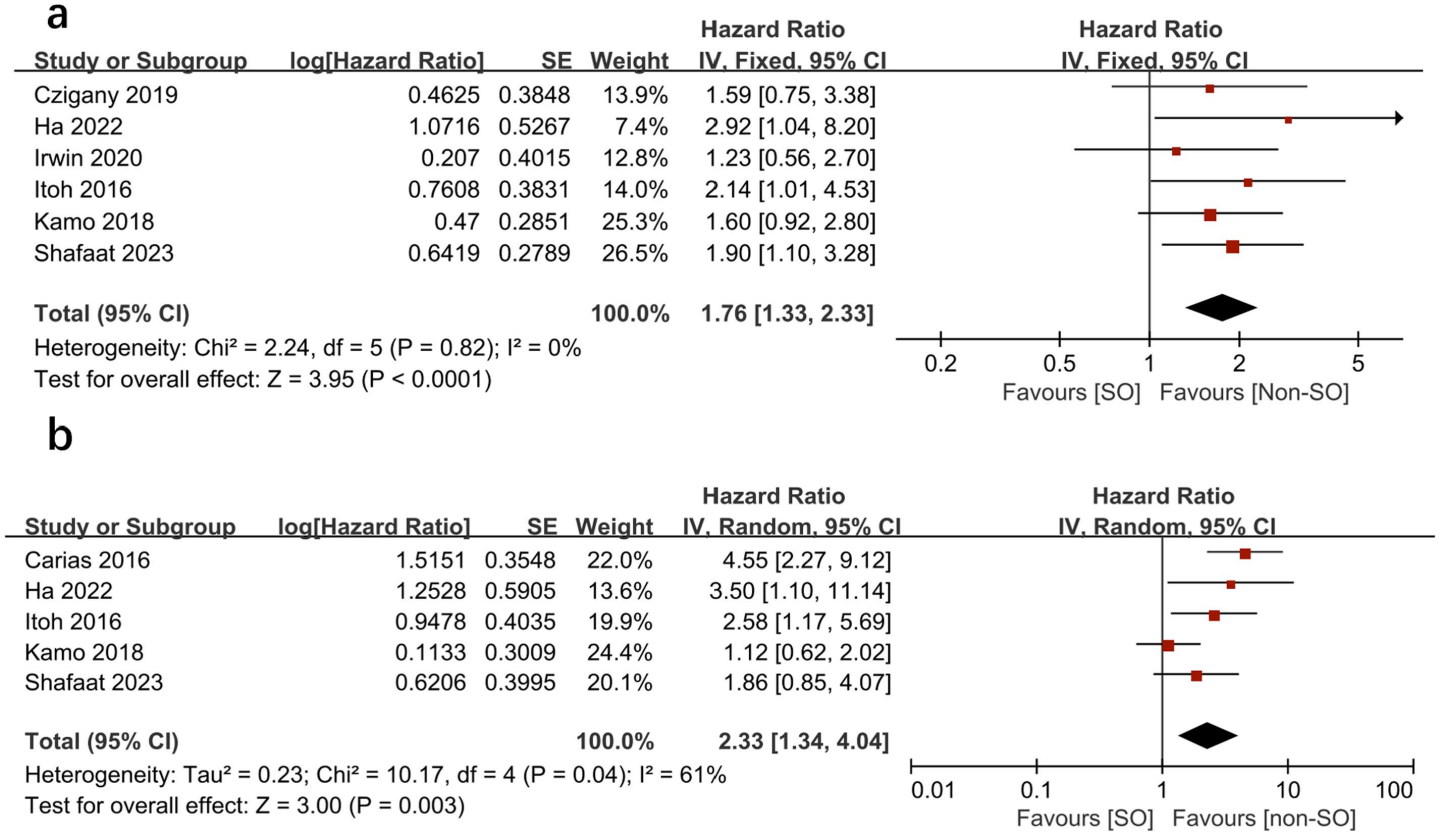
Meta-analysis of univariate results **(A)** and multivariate results **(B)** reporting the impact of sarcopenic obesity on mortality in patients undergoing liver transplantation by categorical variable.

A total of seven studies reported the outcome of overall survival as hazard ratios (HRs) in patients with pre-transplant SO (16–19, 25, 26, 28). When pooled, patients with SO had a significantly increased risk of mortality compared to patients without SO in both univariate analysis (HR = 1.76, 95% CI 1.33–2.33, *I*^2^ = 0%, *p* < 0.0001) (16–19, 25, 26) (Figure 2A) and multivariate analysis (HR = 2.33, 95%CI 1.34 to 4.04, *I*^2^ = 61%, *p* = 0.003) (16, 18, 25, 26, 28) as a categorical variable (Figure 2B). This indicates that SO has an independent significant prognostic effect on overall survival.

**Figure 3:**
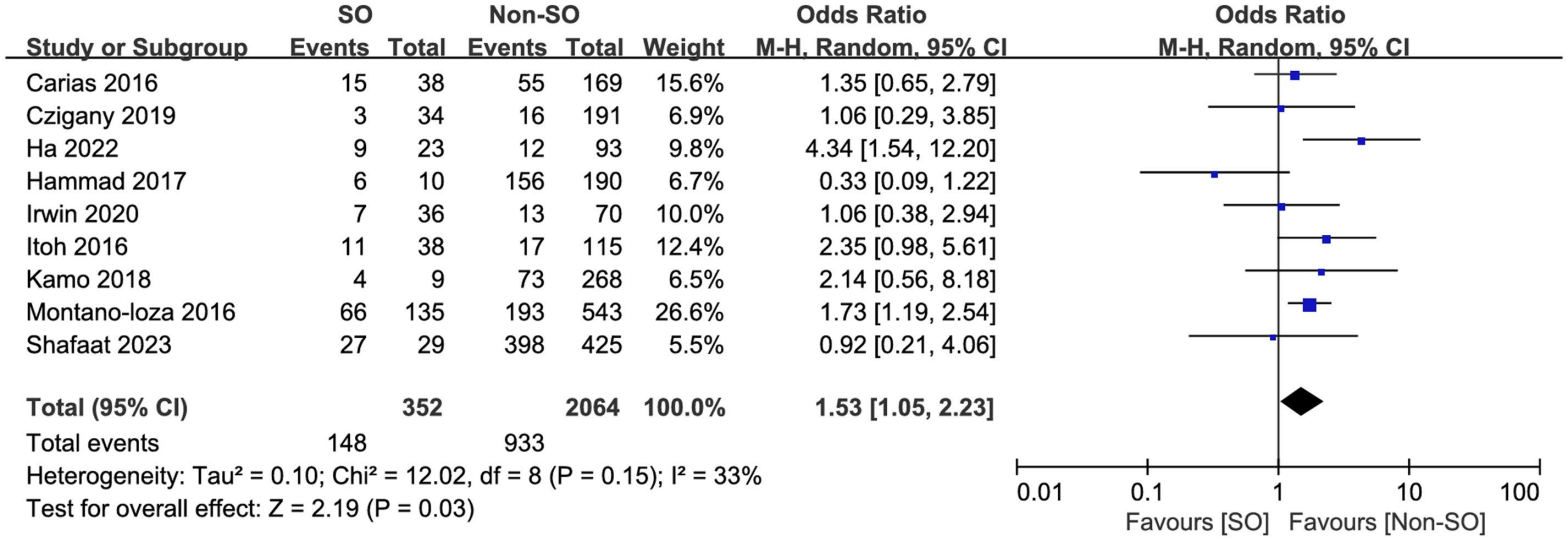
Risk ratios of mortality with sarcopenic obesity vs. non-sarcopenic obesity in mortality rate at the longest follow-up available.

**Figure 4:**
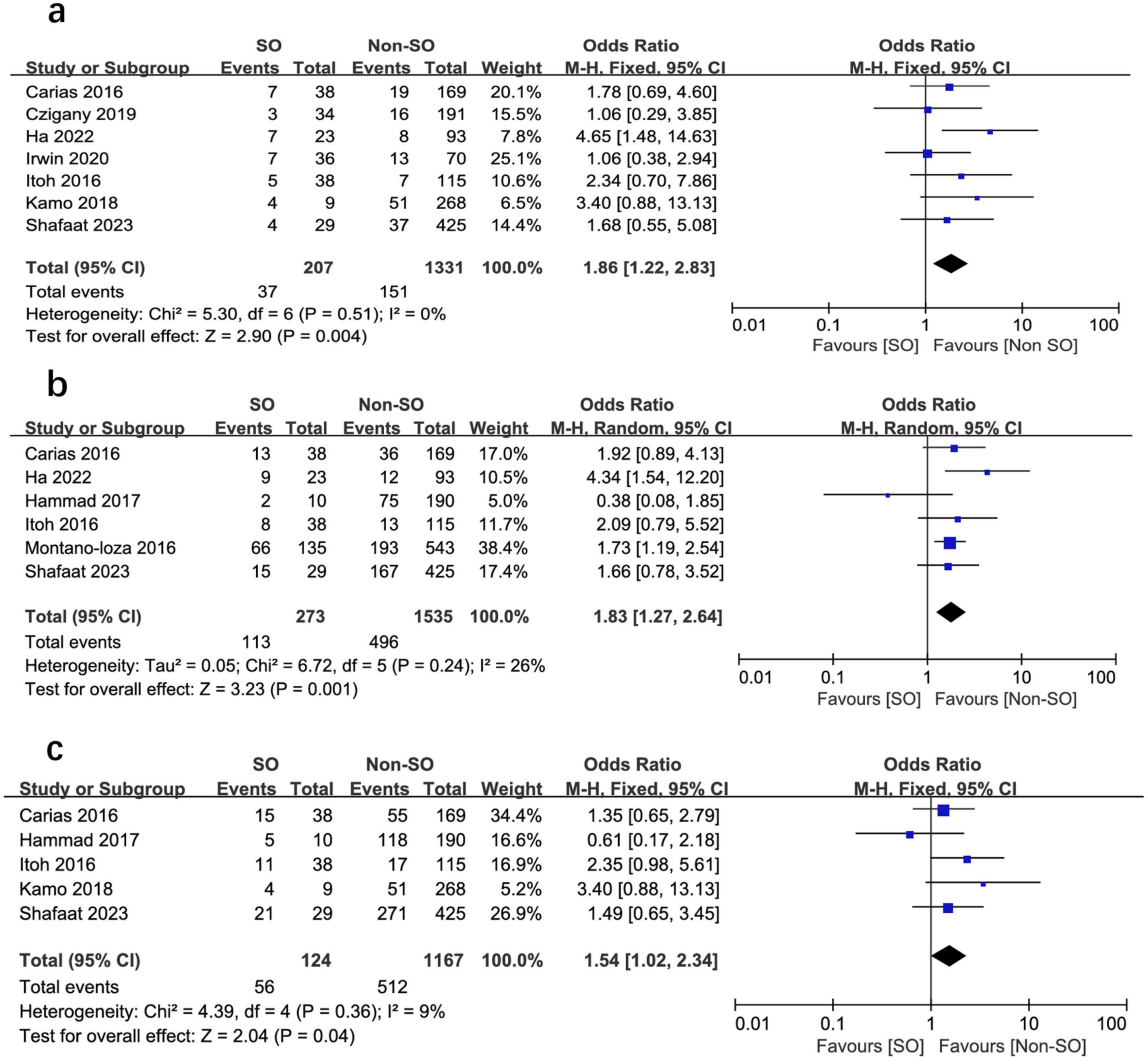
Meta-analysis of results reporting risk ratios of mortality with sarcopenic obesity vs. non-sarcopenic obesity in 1-year mortality **(A)**, 3-year mortality **(B)**, and 5-year mortality **(C)**.

**Figure 5:**
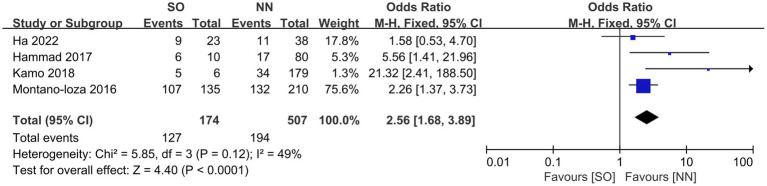
Meta-analysis of results reporting the impact of sarcopenia **(A)** and sarcopenic obesity **(B)** on mortality as a continuous variable in studies that report both findings in the same study cohort.

All the included studies compared mortality in SO patients with all the other recipients. Overall, SO was associated with higher mortality at the longest follow-up available than non-SO patients (OR = 1.53, 95% CI 1.05–2.23, *I*^2^ = 33%, *p* = 0.03) (16–19, 24–28) (Figure 3). Similar results were also found when different follow-ups were considered, including 1-year mortality (OR = 1.86, 95% CI 1.22–2.83, *I*^2^ = 0%, *p* = 0.004, Figure 4A), 3-year mortality (OR = 1.83, 95% CI 1.27–2.64, *I*^2^ = 40%, *p* = 0.001, Figure 4B), and 5-year mortality (OR = 1.54, 95% CI 1.02–2.34, *I*^2^ = 9%, *p* = 0.04, Figure 4C). Moreover, the pooled results of four studies comparing patients with SO and non-sarcopenic/non-obesity (NN) patients showed that SO significantly increased mortality at the longest follow-up (OR = 3.10, 95% CI 1.43–6.75, *I*^2^ = 49%, *p* = 0.004) (18, 24, 25, 27) (Figure 5). We proceeded to perform subgroup analyses based on of SO definition type (SMI + BMI or VFA + SMI) and found that the majority of the subgroup analyses confirmed significantly higher mortality in patients with SO (Table 2).

**Table 2:** Subgroup analysis on the primary outcome of mortality.

Subgroup	variables	Studies number	Patient number	Event in mortality group	Event in control group	HR (95% CI)	*I*^2^ (%)	*P*
BMI + SMI	HR by univariate analysis	3		–	–	1.63 [1.15, 2.31]	0	0.006
	HR by multivariate analysis	2		–	–	1.35 [0.84, 2.18]	3	0.22
	Longest follow-up available	6	1922	126/254	885/1668	1.30 [0.73, 2.31]	54	0.38
	1-year	4	1,044	21/109	121/935	1.62 [0.93, 2.82]	0	0.09
	3-year	4	1,539	96/212	471/1327	1.62 [1.20, 2.19]	15	0.002
	5-year	4	1,138	46/83	514/1055	1.48 [0.92, 2.38]	51	0.11
SMI + VFA	HR by univariate analysis	4		–	–	1.51 [1.05, 2.17]	35	0.03
	HR by multivariate analysis	3		–	–	3.54 [2.20, 5.70]	0	<0.00001
	Longest follow-up available	4	771	27/104	118/667	2.32 [1.37, 3.93]	0	0.002
	1-year	4	771	19/104	82/667	2.46 [1.35, 4.48]	2	0.03
	3-year	2	269	17/61	25/208	2.91 [1.44, 5.86]	2	0.03
	5-year	2	430	15/47	68/383	2.60 [1.25, 5.40]	0	0.01

BMI, body mass index; HR, hazard ratio, SMI, skeletal muscle index; VFA, visceral fat area.

**Figure 6:**
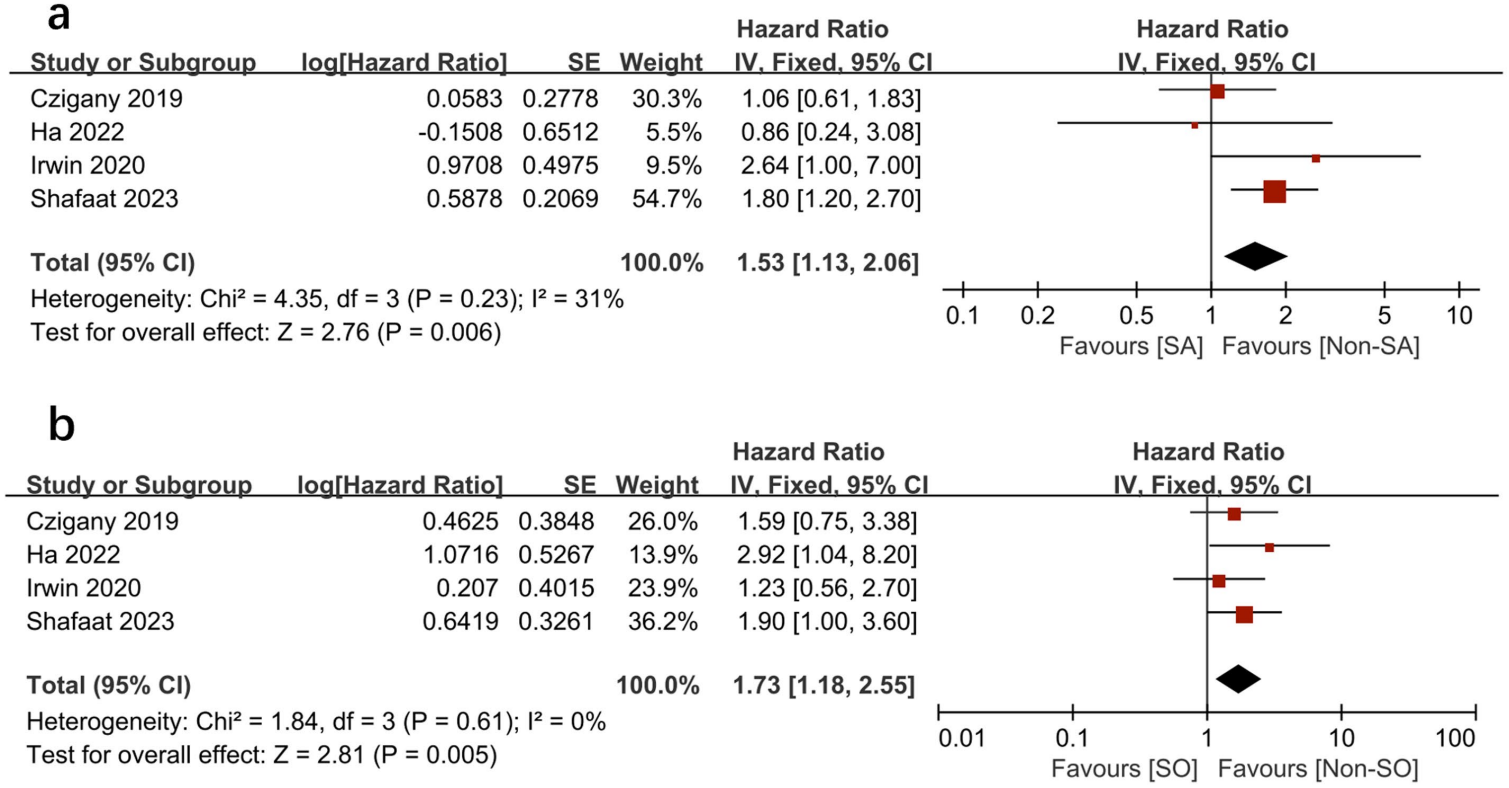
Meta-analysis of univariate results reporting the impact of sarcopenia **(A)** and sarcopenic obesity **(B)** on mortality.

**Figure 7:**
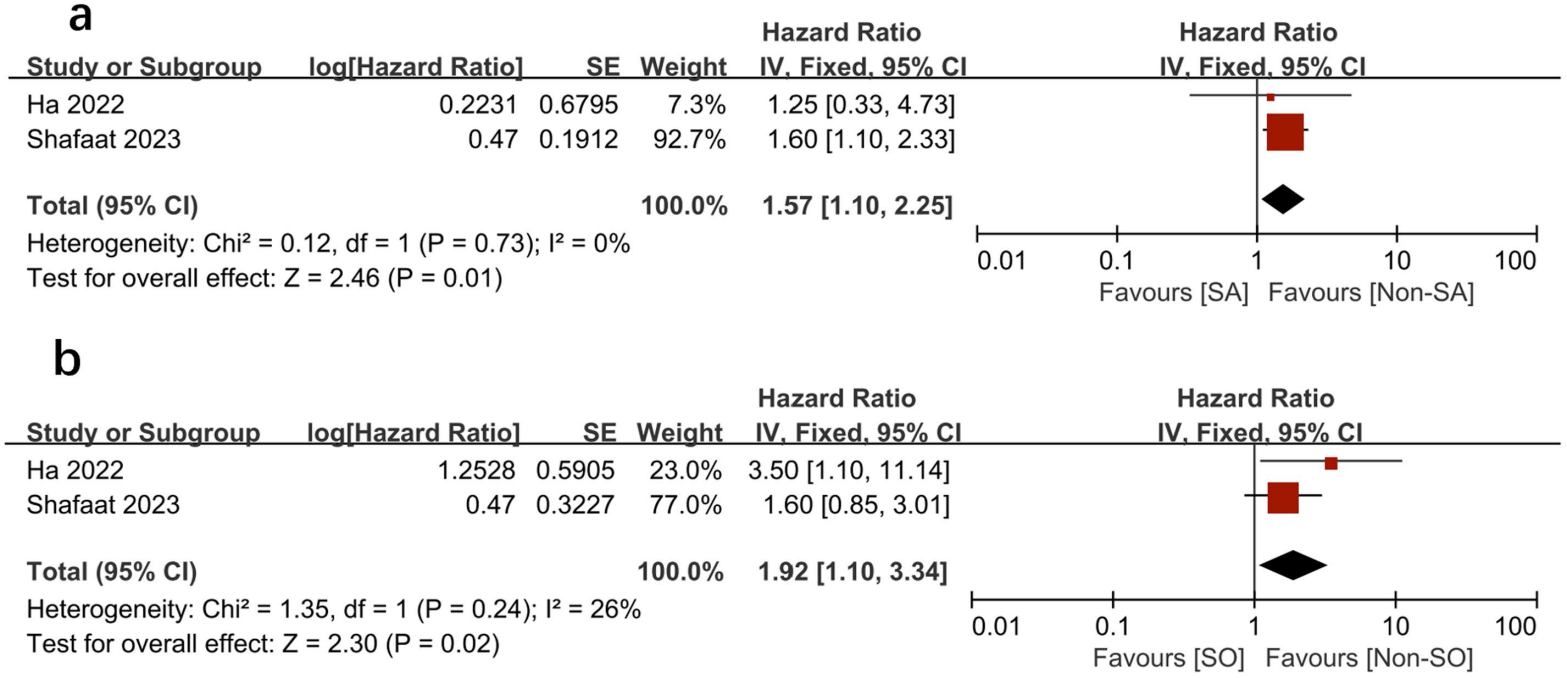
Meta-analysis of multivariate results of the impact of sarcopenia **(A)** and sarcopenic obesity **(B)** on mortality.

We further compared the effects of sarcopenia and SO on survival by analyzing the HRs for each, as reported in the same study cohorts. We found that sarcopenia and SO had independent negative effects on survival. Figures 6, 7 show the effect of sarcopenia and SO on survival using univariate values (sarcopenia: HR = 1.53, 95% CI 1.13–2.06, *I*^2^ = 31%, *p* = 0.006 vs. SO: HR = 1.73, 95%CI 1.18–2.55, *I*^2^ = 0%, *p* = 0.005) (16–19) and multivariate values (sarcopenia: aHR = 1.57, 95% CI 1.10–2.25, *I*^2^ = 0%, *p* = 0.01 vs. SO: aHR = 1.92, 95%CI 1.10–3.34, *I*^2^ = 26%, *p* = 0.02) (16, 18).

In addition, the assessment of publication bias using visually inspecting funnel plots showed no potential publication bias in the included studies (Supplementary File 4).

## 4 Discussion

In the current meta-analysis, we included nine studies with 2,416 adults and showed that pre-LT patients commonly experience SO, with a mean prevalence as high as 34%. In addition to sarcopenia, pre-transplant SO is a robust independent predictor of mortality in this patient population. Overall, SO was associated with a more than 130% increase in mortality risk (HR = 2.33, 95%CI 1.34–4.04). Further analyses of mortality rates between patients with and without SO at different follow-ups confirmed this finding.

### 4.1 Our results in comparison with previous reviews

Our meta-analysis provides strong evidence to support a previously published meta-analysis on this topic (13), i.e., that SO worsens survival after LT. However, the previous authors only included three studies with a total of 637 patients in the final analyses, and in one of the included studies they entered incorrect data on patients with SO, which significantly reduced the robustness of their results (13). Therefore, on this basis, we included six additional studies with a total of 2,416 cases, which had adequate statistical power to fully assess the risk of mortality. Thus, we were able to summarize not only the mortality results between groups but also the results of univariate and multivariate regression analyses reported by the included studies, which helps reduce the impact of clinical heterogeneity on our findings. In addition, we compared the effects of SO and sarcopenia on survival and confirmed the independent prognostic significance of SO. Interestingly, our study observed that SO was superior to sarcopenia alone in predicting survival after LT, as shown by analyses of pooled unadjusted results (HR:1.73 vs. 1.53) and pooled adjusted results (HR:1.92 vs. 1.57). This indicates that muscle quality characterized by SO might even bear a higher independent prognostic value in predicting survival after LT than sarcopenia.

Additionally, our findings are consistent with recent meta-analyses of other patient populations, including gastrointestinal oncology, emergency laparotomy, and cirrhotic patients (29–31), which have highlighted adverse clinical outcomes with SO. As a result, our study adds a new population to the body of evidence.

### 4.2 Explain the results of our research

Emerging studies have shown that the muscle–liver–adipose tissue axis has received significant attention as a major endogenous factor in SO (32). This involves a complex array of pathological mechanisms, including systemic and muscular oxidative stress, inflammation, muscle anabolic resistance and insulin resistance, and metabolic lipotoxicity due to ectopic lipid deposition (33–35). The result is reduced muscle mass and a severe imbalance between protein synthesis and protein catabolism. Notably, visceral fat accumulation releases various pro-inflammatory adipokines, such as leptin, TNF-*α*, interleukin (IL)-1, and IL-6, C-reactive protein, and decreases lipocalin or IL-15, thereby affecting the metabolism, function, and immune system of the skeletal muscle tissue (34, 36). In addition, intestinal dysbiosis appears to adversely affect skeletal muscle health and liver function via the gut–liver–muscle tissue axis, which may contribute to skeletal muscle dysfunction with its induced inflammation and mitochondrial dysfunction (37). Further studies are needed in the future to confirm the detailed mechanisms.

### 4.3 Current literature and future research

The definition of obesity in patients with SO warrants further investigation. The studies included in our analysis predominantly defined SO as the combination of low SMI and high BMI (16, 17, 24, 25, 27, 28), rather than high VFA (18, 19, 25, 26). Notably, our subgroup analyses indicated that both definitions were correlated with unfavorable outcomes in LT patients; however, discrepancies persisted in the incidence and prognostic significance of these two distinct definitions (25, 38). In individuals with end-stage liver disease and cirrhosis, elevated BMI may not accurately reflect body composition due to factors such as peripheral edema, ascites, and abdominal fluid overload. Consequently, some experts propose defining SO as a syndrome typified by reduced muscle mass and increased VFA on CT scans, advocating against the use of BMI as the sole criterion for obesity in this context (39).

The assessment of muscle mass at the L3 vertebra typically involves measuring the total skeletal muscle area, with the measurement of the psoas muscle area being another method. The latter method is less recommended due to the psoas muscle’s limited representation of overall sarcopenia. Further research is essential to validate the disparities arising from these two measurement approaches.

Our study’s findings contradict the established “obesity paradox,” which suggests that obesity is associated with numerous comorbidities but is a potential protective factor for mortality (14). The results of our combined regression analysis in this study revealed that muscle mass characterized by SO holds an independent prognostic value in predicting survival after LT. Understanding the importance of this is crucial because overweight or obese patients may mask the decline in muscle atrophy, leading to the oversight of findings in patients with sarcopenia. This is particularly significant in the context of an aging society and an increasingly obese population. For example, the study included patients with a high prevalence of obesity, reaching up to 45% (ranging from 22 to 60%), and a high prevalence of SO, reaching up to 32.4% (16.9–52.3%) (Table 1).

Identifying promising interventions to improve muscle function and thus survival outcomes is necessary due to the relationship between pre-LT SO and poor prognosis. Various trials have intensively studied preventive and therapeutic measures for sarcopenic obesity. Methods to improve skeletal muscle content and function include physical activity, such as resistance training (40) and aerobic endurance training; nutritional regimens, such as energy restriction (low-calorie, low-fat, and low-carbohydrate diets) (41), high-protein supplements, and branched-chain amino acids; and pharmacological treatments, such as ammonia-lowering treatments, which can enhance skeletal muscle performance and function (42–44). Recent guidelines recommend combining evidence-based nutritional therapy and protein supplementation with exercise (45).

### 4.4 Limitations

Several limitations of this study should be acknowledged. First, it is important to note that this review included retrospective studies, which may have influenced our meta-analysis with the same biases found in the original study. Second, although we used regression methods and sensitivity analyses to explore sources of heterogeneity, we were unable to consider all influencing factors. Many studies reported only a few predefined outcomes, particularly secondary outcomes, which reduced the robustness of the combined analyses. These factors include severity, duration, and treatments used for obesity; the severity of sarcopenia; the type of diet; and the use of supplements, all of which might favor heterogeneity among the studies, making direct comparisons challenging. Additionally, there was a lack of consensus on thresholds for SMI, CT protocols, and definitions of obesity, which affected comparability across studies.

## 5 Conclusion

SO is common among pre-LT recipients, with its prevalence varying. When assessed by CT, SO is a reliable factor influencing overall survival in this patient population. However, it is important to note that the included studies used different definitions of SO, which is the main reason for the existing heterogeneity. In addition, further studies are needed to determine the optimal threshold for sarcopenia and to define CT-assessed SO according to geography, ethnicity, and age to confirm our findings.

## Data Availability

The original contributions presented in the study are included in the article/Supplementary material, further inquiries can be directed to the corresponding authors.

## Footnotes

^1^ https://doi.org/10.37766/inplasy2024.2.0069

^2^https://www.basesearch.net; https://scholar.google.com
