# Supplementary material for: Sarcopenic obesity is significantly associated with poorer overall survival after liver transplantation: a systematic review and meta-analysis
Source: Front Nutr. 2024 Dec 16;11:1387602. doi: 10.3389/fnut.2024.1387602 (PMC11684403; doi:10.3389/fnut.2024.1387602)
Supplement: Supplementary file 1 [file Data_Sheet_1.docx]

**Sarcopenic obesity is significantly associated with poorer overall survival after liver transplantation: a systematic review and meta-analysis**

**Appendix File**

1. Additional file 1

Checklist following PRISMA guideline………………………………………………………………………………………………………...2

2. Additional file 2

Search strategy………………………………………………………………………………………………….….……………………..…..…….…..5

3. Additional file 3

Quality assessment and overall risk of bias of included studies………………….……….…………………………….…….....7

4. Additional file 4

Funnel plot of comparison: mortality………………………………………..……………………………….……………….………………8

**Appendix file 1**

**PRISMA 2009 checklist**

| **Section/topic** | **#** | **Checklist item** | **Reported on page #** |
| --- | --- | --- | --- |
| **TITLE** | | |  |
| Title | 1 | Identify the report as a systematic review, meta-analysis, or both. | 1 |
| **ABSTRACT** | | |  |
| Structured summary | 2 | Provide a structured summary including, as applicable: background; objectives; data sources; study eligibility criteria, participants, and interventions; study appraisal and synthesis methods; results; limitations; conclusions and implications of key findings; systematic review registration number. | 2 |
| **INTRODUCTION** | | |  |
| Rationale | 3 | Describe the rationale for the review in the context of what is already known. | 4 |
| Objectives | 4 | Provide an explicit statement of questions being addressed with reference to participants, interventions, comparisons, outcomes, and study design (PICOS). | 5 |
| **METHODS** | | |  |
| Protocol and registration | 5 | Indicate if a review protocol exists, if and where it can be accessed (e.g., Web address), and, if available, provide registration information including registration number. | 7 |
| Eligibility criteria | 6 | Specify study characteristics (e.g., PICOS, length of follow-up) and report characteristics (e.g., years considered, language, publication status) used as criteria for eligibility, giving rationale. | 7 |
| Information sources | 7 | Describe all information sources (e.g., databases with dates of coverage, contact with study authors to identify additional studies) in the search and date last searched. | 7 |
| Search | 8 | Present full electronic search strategy for at least one database, including any limits used, such that it could be repeated. | 7 and Appendix file 2 |
| Study selection | 9 | State the process for selecting studies (i.e., screening, eligibility, included in systematic review, and, if applicable, included in the meta-analysis). | 7-8 |
| Data collection process | 10 | Describe method of data extraction from reports (e.g., piloted forms, independently, in duplicate) and any processes for obtaining and confirming data from investigators. | 8 |
| Data items | 11 | List and define all variables for which data were sought (e.g., PICOS, funding sources) and any assumptions and simplifications made. | 8 |
| Risk of bias in individual studies | 12 | Describe methods used for assessing risk of bias of individual studies (including specification of whether this was done at the study or outcome level), and how this information is to be used in any data synthesis. | 8 |
| Summary measures | 13 | State the principal summary measures (e.g., risk ratio, difference in means). | 8 |
| Synthesis of results | 14 | Describe the methods of handling data and combining results of studies, if done, including measures of consistency (e.g., I^2^) for each meta-analysis. | 9 |

| Risk of bias across studies | 15 | Specify any assessment of risk of bias that may affect the cumulative evidence (e.g., publication bias, selective reporting within studies). | 8 |
| --- | --- | --- | --- |
| Additional analyses | 16 | Describe methods of additional analyses (e.g., sensitivity or subgroup analyses, meta-regression), if done, indicating which were pre-specified. | 8-9 |
| **RESULTS** | | |  |
| Study selection | 17 | Give numbers of studies screened, assessed for eligibility, and included in the review, with reasons for exclusions at each stage, ideally with a flow diagram. | 10  Figure 1 |
| Study characteristics | 18 | For each study, present characteristics for which data were extracted (e.g., study size, PICOS, follow-up period) and provide the citations. | 10  Table 1 |
| Risk of bias within studies | 19 | Present data on risk of bias of each study and, if available, any outcome level assessment (see item 12). | 10 |
| Results of individual studies | 20 | For all outcomes considered (benefits or harms), present, for each study: (a) simple summary data for each intervention group (b) effect estimates and confidence intervals, ideally with a forest plot. | 10 |
| Synthesis of results | 21 | Present results of each meta-analysis done, including confidence intervals and measures of consistency. | 10-11 |
| Risk of bias across studies | 22 | Present results of any assessment of risk of bias across studies (see Item 15). | Appendix File 3 |
| Additional analysis | 23 | Give results of additional analyses, if done (e.g., sensitivity or subgroup analyses, meta-regression [see Item 16]). | 11-12  Table 2 |
| **DISCUSSION** | | |  |
| Summary of evidence | 24 | Summarize the main findings including the strength of evidence for each main outcome; consider their relevance to key groups (e.g., healthcare providers, users, and policy makers). | 13 |
| Limitations | 25 | Discuss limitations at study and outcome level (e.g., risk of bias), and at review-level (e.g., incomplete retrieval of identified research, reporting bias). | 17 |
| Conclusions | 26 | Provide a general interpretation of the results in the context of other evidence, and implications for future research. | 18 |
| **FUNDING** | | |  |
| Funding | 27 | Describe sources of funding for the systematic review and other support (e.g., supply of data); role of funders for the systematic review. | 19 |

**Appendix file 2**

Database: PubMed, Embase, Cochrane library (Search completed 16th October 2023)

--------------------------------------------------------------------------------------------------------------------------------------------------------------------------------------------------------

**PubMed**

(((((liver transplant[Title/Abstract]) OR (liver transplantation[Title/Abstract])) OR ("Liver Transplantation"[Mesh]))) AND ((((Sarcopenia[Title/Abstract]) OR (sarcopenic[Title/Abstract])) OR ("Sarcopenia"[Mesh])) OR (((((((((((((((((sarcopaenia[Title/Abstract]) OR (sarcopaenic[Title/Abstract])) OR (myopenia[Title/Abstract])) OR (myopaenic[Title/Abstract])) OR (myopenic[Title/Abstract])) OR (myopaenia[Title/Abstract])) OR (body composition[Title/Abstract])) OR (lean body[Title/Abstract])) OR (low muscle mass[Title/Abstract])) OR (low muscle function[Title/Abstract])) OR (frailty[Title/Abstract])) OR (muscle mass[Title/Abstract])) OR (muscle atrophy[Title/Abstract])) OR (muscular atrophy[Title/Abstract])) OR (muscle depletion[Title/Abstract])) OR (core muscle[Title/Abstract])) OR (muscle strength[Title/Abstract])))) AND (((((((obesity[Title/Abstract]) OR (obese[Title/Abstract])) OR (adiposity[Title/Abstract])) OR (adipose[Title/Abstract])) OR (fat mass[Title/Abstract])) OR (overweight[Title/Abstract])) OR ("Obesity"[Mesh]))

-------------------------------------------------------------------------------------------------------------------------------------------------------------------------------------------------------

**Embase**

#4. #1 AND #2 AND #3

#3. ‘obesity’ /exp OR ‘obesity’ : ab,ta, kw OR ‘obese’ : ab,ta, kw OR ‘adiposity’ : ab,ta, kw OR ‘adipose’ : ab,ta, kw OR ‘fat mass’ : ab,ta, kw OR ‘overweight’ : ab,ta, kw

#2. 'liver transplant':ab,ti AND ([embase]/lim OR [medline]/lim) OR 'liver transplantation'/exp OR 'liver transplantation':ab,ti AND ([embase]/lim OR [medline]/lim)

#1. ‘sarcopenia’ /exp OR‘sarcopenia’ OR‘intermuscular adipose tissue’OR‘muscle attenuation’ OR‘muscle composition’ /exp OR‘muscle fat infiltration’:ab, ta, kw OR‘myosteatosis’/exp OR‘intramuscular fat’ : ab,ta, kw OR‘muscle density’/exp OR‘myosteatosis’OR‘myopenia’ OR‘myopaenic’OR myopenic’OR‘myopaenia’OR ‘body composition’OR ‘lean body’OR ‘low muscle mass’OR ‘low muscle function’OR‘frailty’OR‘muscle mass’OR‘muscle atrophy’OR‘muscular atrophy’OR‘muscle depletion’OR‘core muscle’OR‘muscle strength’

------------------------------------------------------------------------------------------------------------------------------------------------------------------------------------------------------

**Cochrane library**

ID Search

#1 MeSH descriptor: [liver transplantation] explode all trees

#2 ("liver transplantation "):ti,ab,kw (Word variations have been searched)

#3 (liver transplant):ti,ab,kw (Word variations have been searched)

#4 #1 OR #2 OR #3

#5 ("sarcopenia"):ti,ab,kw (Word variations have been searched)

#6 ("ntermuscular adipose tissue"):ti,ab,kw (Word variations have been searched)

#7 ("muscle composition"):ti,ab,kw (Word variations have been searched)

#8 ("myopenia"):ti,ab,kw (Word variations have been searched)

#9 ("myopaenic"):ti,ab,kw (Word variations have been searched)

#10 ("myopenic"):ti,ab,kw (Word variations have been searched)

#11 ("myopaenia"):ti,ab,kw (Word variations have been searched)

#12 ("body composition"):ti,ab,kw (Word variations have been searched)

#13 ("lean body"):ti,ab,kw (Word variations have been searched)

#14 ("low muscle mass"):ti,ab,kw (Word variations have been searched)

#15 ("low muscle function"):ti,ab,kw (Word variations have been searched)

#16 ("frailty"):ti,ab,kw (Word variations have been searched)

#17 ("muscle mass"):ti,ab,kw (Word variations have been searched)

#18 ("muscle atrophy"):ti,ab,kw (Word variations have been searched)

#19 ("muscular atrophy"):ti,ab,kw (Word variations have been searched)

#20 ("muscle depletion"):ti,ab,kw (Word variations have been searched)

#21 ("core muscle"):ti,ab,kw (Word variations have been searched)

#22 ("muscle strength"):ti,ab,kw (Word variations have been searched)

#23 #5 OR #6 OR #7 OR #8 OR #9 OR #10 OR #11OR #12 OR #13 OR #14 OR #15 OR #16 OR #17 OR #18 OR #19 OR #20 OR #21 OR #22

#24 ("obesity"):ti,ab,kw (Word variations have been searched)

#25 ("obese"):ti,ab,kw (Word variations have been searched)

#26 ("adiposity"):ti,ab,kw (Word variations have been searched)

#27 ("adipose):ti,ab,kw (Word variations have been searched)

#28 ("fat mass"):ti,ab,kw (Word variations have been searched)

#29 ("overweight"):ti,ab,kw (Word variations have been searched)

#30 #24 OR #25 OR #26 OR #27 OR #28 OR #29

#31 #4 OR #23 OR #30

---------------------------------------------------------------------------------------------------------------------------------------------------------------------------------------------------------

Web of Science

1. liver transplant [topic]

2. liver transplantation [topic]

3. or/1-2

4. obesity [topic]

5. obese [topic]

6. adiposity [topic]

7. adipose [topic]

8. overweight [topic]

9. or/4-8

10. sarcopaenic [topic]

11. myopenia [topic]

12. myopaenic [topic]

13. myopenic [topic]

14. low muscle mass [topic]

15. low muscle function [topic]

16. frailty [topic]

17. muscle atrophy [topic]

18. muscular atrophy [topic]

19. muscle depletion [topic]

20. core muscle [topic]

21. muscle strength [topic]

22. or/10-22

23.or/10-11

24. 3 and 9 and 22

------------------------------------------------------------------------------------------------------------------------------------------------------------------------------------------------------

Wanfang database

主题：（肥胖 or 超重 or ）and 主题：（肝移植 or 肝脏移植）and 主题：（肌肉减少症 or 虚弱 or 肌肉萎缩 or 肌肉力量 or 肌肉减少 or 少肌症）

----------------------------------------------------------------------------------------------------------------------------------------------------------------------------------------------------

China National Knowledge Infrastructure database

TKA=“肝脏移植” and TKA=(“肥胖” + “超重” + “体重过高”）and TKA=(“肌肉减少症” or “虚弱” or “肌肉萎缩” or “肌肉力量” or “肌肉减少” or ”少肌症“）


**Appendix file 3**

**Summary Risk of Bias in assessment with overall study-level risk of bias.**


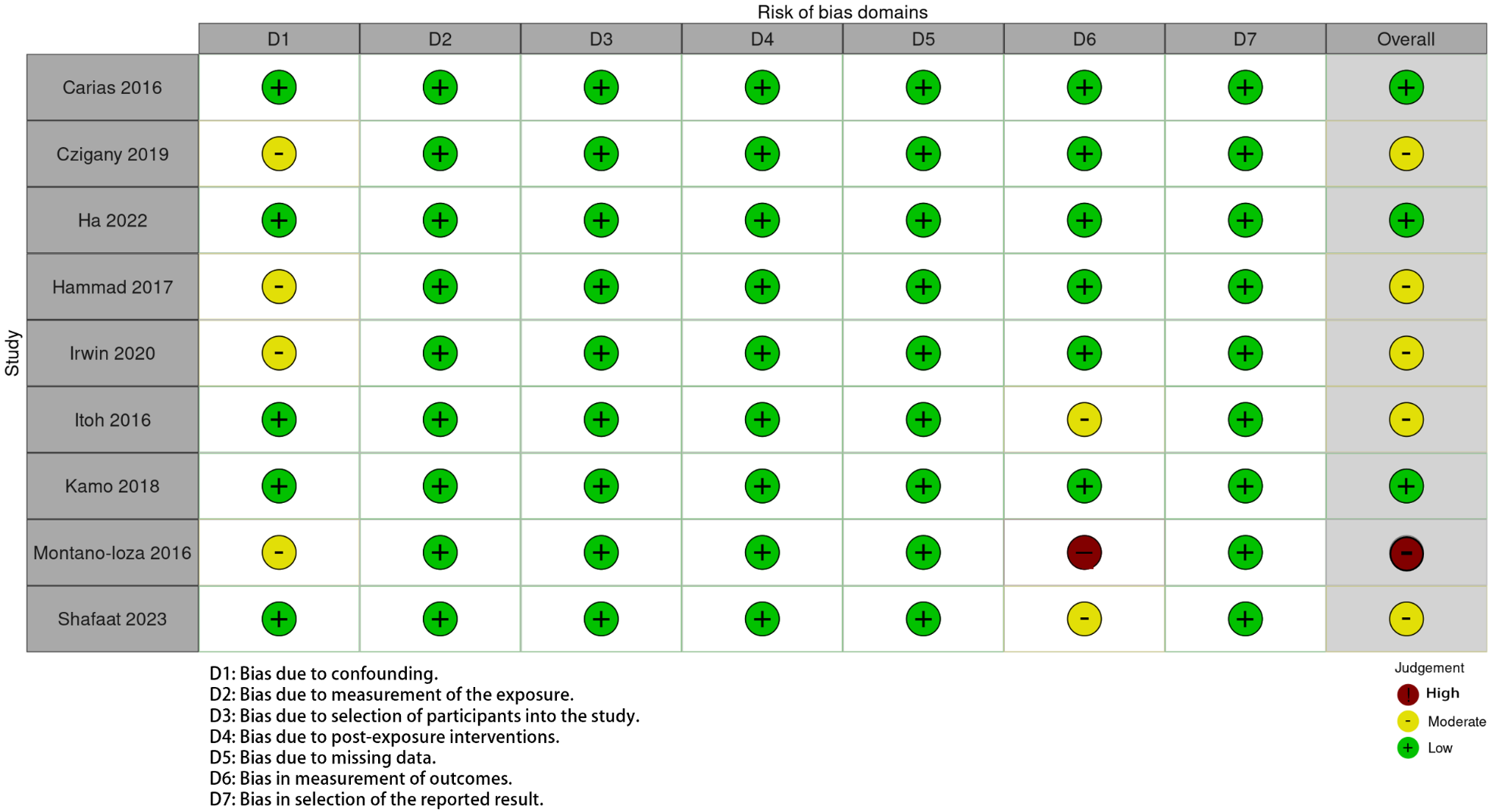


**Appendix file 4**


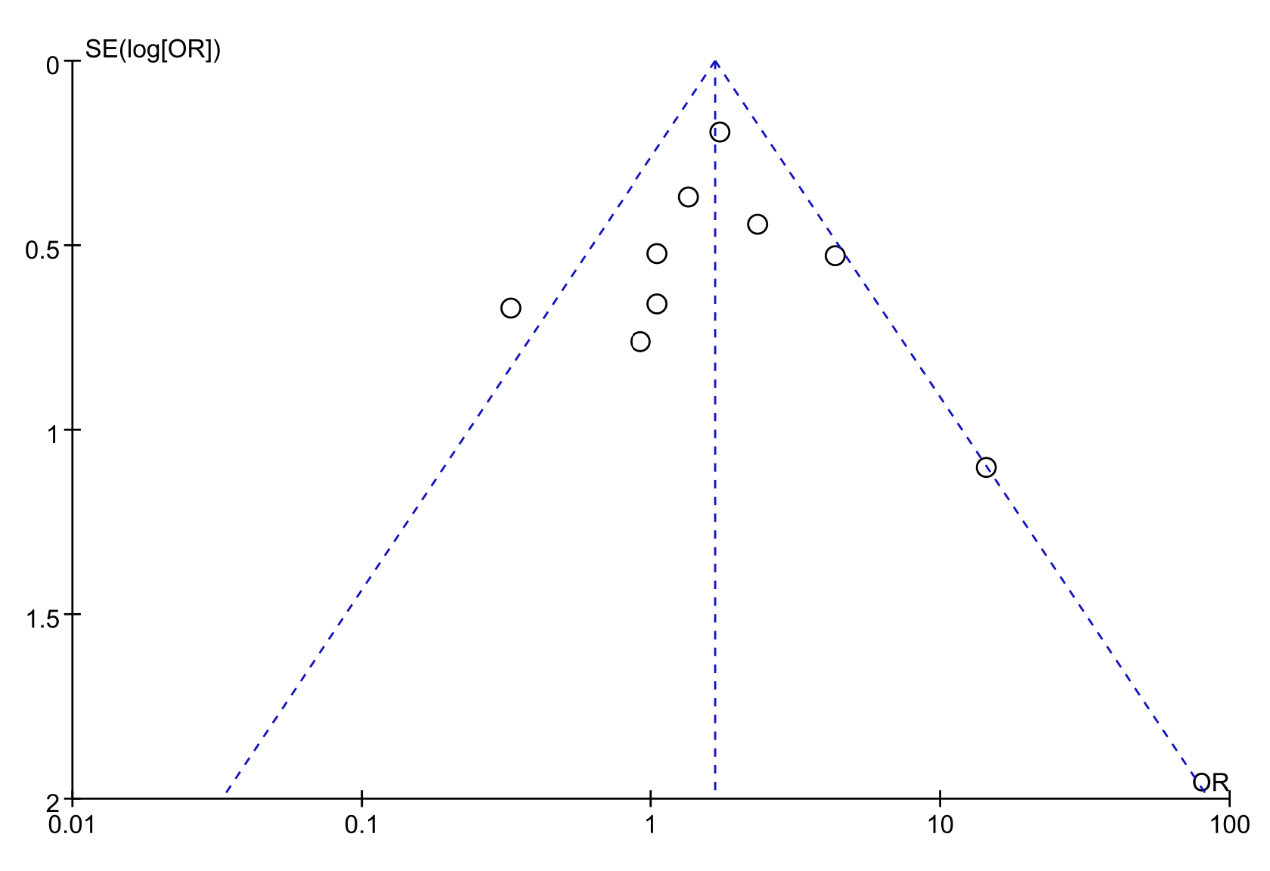


**Figure S6: Publication bias （outcome of between groups mortality, 9 studies）**
